# Gastrostomy Tube Placement by Radiological Methods for Older Patients Requiring Enteral Nutrition: Not to be Forgotten

**DOI:** 10.3389/fmed.2018.00274

**Published:** 2018-09-26

**Authors:** Vered Hermush, Yitshal Berner, Yael Katz, Yanina Kunin, Irena Krasniansky, Yael Schwartz, Debbie Mimran Nahon, Ana Elizariev, Gad Mendelson

**Affiliations:** ^1^Department of Geriatrics and Skilled Nursing, Laniado Medical Center, Netanya, Israel; ^2^Ruth and Bruce Rappaport Faculty of Medicine, Technion—Isreal Istitute of Technology, Haifa, Israel; ^3^Department of Geriatric Medicine, Meir Medical Center, Kfar Saba and Sackler Medical School, Tel Aviv University, Kfar Saba, Israel; ^4^Dorot Geriatric and Rehabilitation Center, Netanya, Israel

**Keywords:** elderly, enteral nutrition, percutaneous radiological gastrostomy, survival, feeding tube

## Abstract

**Background:** The use of gastrostomy tubes for long-term nutritional support in older patients is frequent. Percutaneous gastrostomy tube placement may be performed using various techniques, including endoscopic, surgical, and radiologically-guided methods. While percutaneous endoscopic gastrostomy (PEG) placement is the most widely used and accepted approach, experience with the use of percutaneous radiological gastrostomy (PRG) is more limited.

**Objective:** To evaluate the safety and short-term outcomes of PRG in older patients requiring long-term enteral feeding.

**Method:** We performed a prospective study involving all patients aged 65 years and older who underwent PRG insertion at the Laniado hospital over a period of 2 years. Adverse events related to the gastrostomy tube insertion were recorded over a period of 3 months following the procedure.

**Results:** A total of 58 patients were included with a mean age of 78.1 years, and 48% were women. The most frequent indications for enteral feeding were stroke (47%) and dementia (41%). The technical success rate was 100% with no immediate procedure-related mortality or morbidity. One-month mortality was 3%, and overall mortality at 3-month follow-up was 16%. Complications were reported in 39 (67%) of patients, with 17 (29%) experiencing more than 1 complication. While most complications (88%) were minor, major complications occurred in 19 (33%) of the patients. Peritonitis was the cause of death in 2 patients, and tube dislodgment occurred in 17 subjects. During the follow-up period 17 (29%) of patients were re-admitted to hospital, with the cause for re-hospitalization being unrelated to the PRG in half of the cases. Neither bleeding nor deep wound infection was detected in the study group.

**Conclusions:** PRG is relatively safe and effective for gastrostomy placement in older patients, and this technique may be of value in patients with oral infections and those receiving anti-thrombotic therapy.

## Introduction

Patients with neurologic dysfunction are at an increased risk for malnutrition due to a combination of cognitive, behavioral and mechanical problems. Dysphagia, with the consequent risk of airway obstruction, dehydration, weight loss, salivary dribbling, and aspiration pneumonia (1), is the most common indication for the initiation of enteral nutrition (2). The primary aim of enteral tube feeding is to provide adequate nutritional support and thus avoid further loss of body weight, correct significant nutritional deficiencies, rehydrate the patient, and arrest the related deterioration in quality of life of the patient resulting from inadequate oral nutrition intake (3).

A variety of options exist for the provision of enteral feeding, and these include nasogastric feeding tubes, percutaneous endoscopic gastrostomy (PEG) tube placement, percutaneous radiological gastrostomy (PRG) and per-oral image-guided gastrostomy (4, 5). Enteral access can also be obtained surgically, but since the advent of less invasive techniques, surgical gastrostomy is more rarely performed (6). In those cases where endoscopic access is difficult to achieve for factors such as technical considerations and/or local availability, the use of radiological or surgical gastrostomy is still relevant (7, 8).

The aim of this study was to evaluate the outcome of PRG tube placement at a single acute-care medical center regarding tube-related morbidity and mortality over a 3-month period of follow-up to determine whether this procedure remains a reasonable alternative for enteral feeding in older people.

## Materials and methods

We performed a prospective study involving all patients aged 65 years and older who underwent PRG insertion during the period 1st July 2014 to 31st July 2016. Eligible patients treated in the geriatric and skilled-nursing care wards at the Laniado Hospital in Netanya underwent PRG insertion in the radiology department at this hospital. Follow-up continued for a period of 3 months at Laniado Hospital and at the Dorot Geriatric and Rehabilitation Center in Netanya.

**Figure 1:**
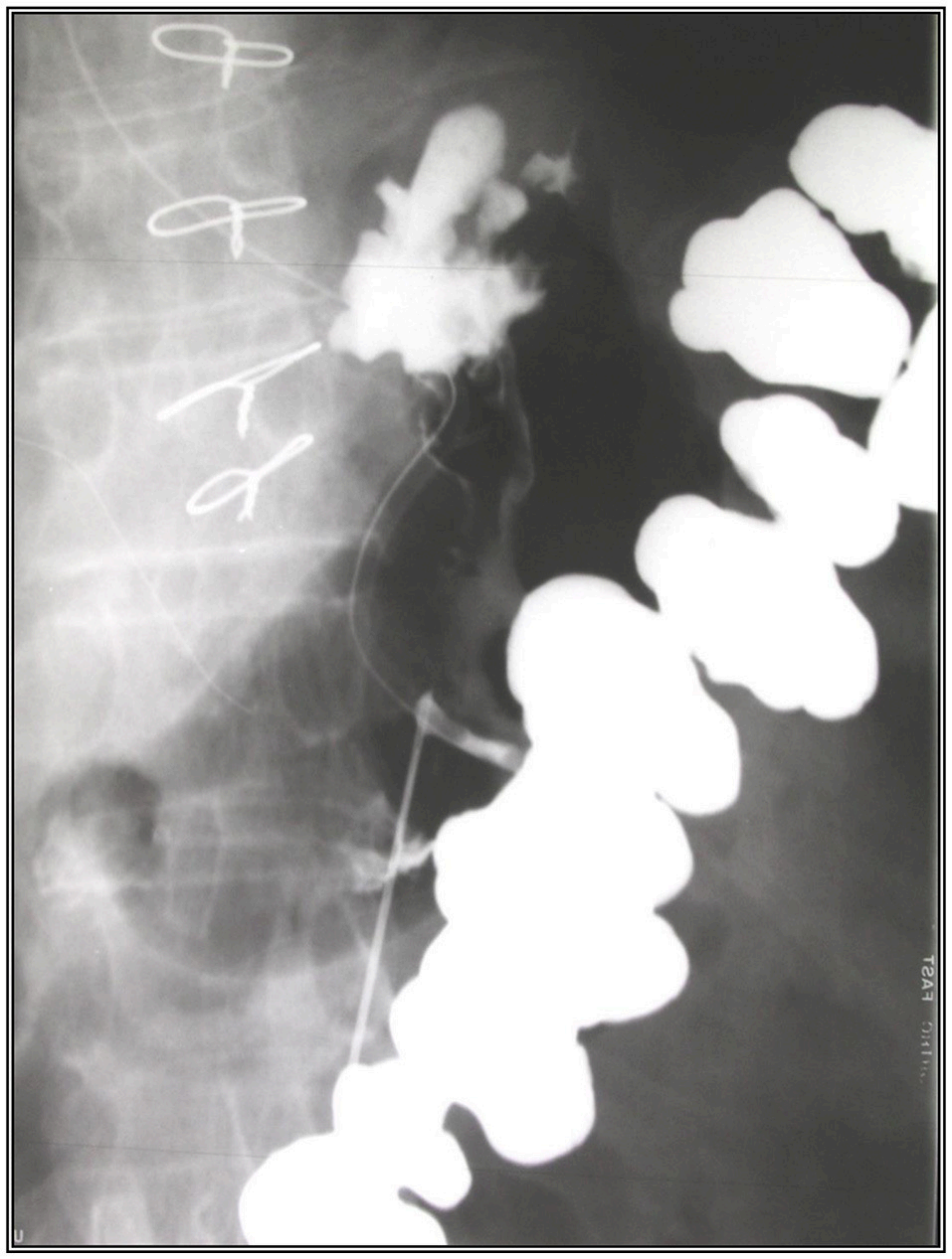
Percutaneous radiological gastrostomy tube insertion.

**Figure 2:**
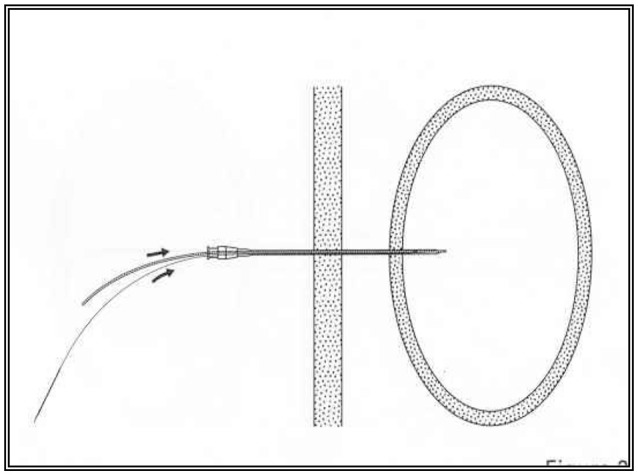
A schematic description of the percutaneous radiological gastrostomy procedure.

In preparation for the insertion of the PRG in the radiology department, an abdominal scan was performed to delineate the hepatic border and thus prevent injury to the liver during the procedure. A nasogastric tube was inserted prior to the procedure to enable the inflation of the stomach. Following the administration of local anesthesia to the abdominal wall, a needle was inserted under radiological control into the gastric lumen and contrast material injected into the stomach to verify that the needle was correctly placed. After a guide was passed via the needle, the needle was removed and the gastrostomy tube (diameter 14.0 Fr, Cook Co.) inserted over the guide as shown in Figures 1, 2.

Eligibility criteria included all patients aged 65 years and older who underwent initial PRG insertion for nutritional support. The study was approved by the Ethics Committee of the Laniado Hospital (0065-13-LND) and the subjects or their legal proxies provided written informed consent for participation in the study.

Data was collected from the medical records and the endoscopic and radiological reporting database, including demographics (age, sex), comorbidity status, indication for insertion, procedural related data, peri- and post-procedural complications, tube dislodgement, tube discontinuation and death.

Patient data included the medical history (particularly cardiac and cerebrovascular diseases, diabetes mellitus, renal diseases, arthritis), physical findings and laboratory data. Nutritional status was assessed by the Mini-Nutritional Assessment (MNA-SF) (9).

The protocol was identical for all the patients: before the procedure, complete history was obtained, and a pertinent physical examination assured the appropriateness for gastrostomy (absence of abdominal hernias, or cutaneous scars). All procedures were monitored by analyzing indications, patient selection, duration of the procedures, and mortality. Conforming with the European Society of Parenteral and Enteral Nutrition (ESPEN) guidelines (2) no standardized antibiotic prophylaxis was provided before PRG was performed. Thrombocyte aggregation inhibitors were not discontinued before PRG, nor were treatments with unfractionated heparin.

The presence of an anesthesiologist in the angiographic room was required and the vital parameters were monitored. Technical success was accomplished when the gastrostomy tube was effectively placed into the stomach and the correct function of the feeding tube was achieved (10).

All complications related to PRG insertion were reported for a period of 3 months following the procedure. The complications were grouped into minor and major complications. The minor complications included peristomal wound infection (reddening with discharge or discharge alone) which may require local antibiotic treatment. The major complications included death, peristomal bleeding, peritonitis, aspiration-related pneumonia or pneumonitis, tube migration and the buried bumper syndrome, and peristomal deep wound infection requiring systemic antibiotic treatment.

## Statistical analysis

Statistical analysis was performed using SPSS 13 (SPSS, Inc., Chicago, IL). Laboratory data are presented as mean ± SD. In all cases normality was assessed by the Kruskal-Wallis test. The difference in the measurements between the first and last measurements (Delta [Δ] change) was calculated and used in analysis between groups on conclusion. *P* values lower than 0.05 levels were considered significant. A chi-square test of 3.84 with one degree of freedom corresponds to a *P* value of 0.05. The Yates correction for chi-square was performed if any of the cells in a 2 X 2 table was <5, or if the summary of the table was <30.

## Results

The study population consisted of 58 patients with a mean age of 78.12 years, and 48% of the subjects were female. Female patients were older (81.76 ± 10.12) than males (74.70 ± 11.72, *p* = 0.007). The leading diagnoses requiring a PRG insertion were stroke or cerebrovascular disease (47%) followed by dementia (41%; Table 1). In 31% of the patients, weight loss was reported in the year prior to the procedure.

**Table 1:** General characteristics of the study patients *N* = 58.

	**Mean ± SD/*N*(%)**
Age (years)	78.12 ± 11.45
Female	28 (48%)
Dementia	24 (41%)
CVA*	27 (47%)
Pulmonary diseases	7 (12%)
Mechanical ventilation	22 (38%)
DM^†^	11 (19%)
Renal failure	3 (5%)
MNA-SF^‡^	5.80 ± 2.56
BMI^§^ kg/m^2^	24.39 ± 5.35
Albumin g/dL	3.17 ± 0.53
Hemoglobin g/dL	10.67 ± 1.83
WBC^||^ cells/μL	9.96 ± 3.18

* *CVA, cerebrovascularaccident*,

† *DM, diabetesmellitus*,

‡ *MNA-SF, Mini-NutritionalAssessmentshort-form*,

§ *BMI, bodymassindex*,

|| *WBC, whitebloodcells*.

There were no anesthetic related intra-procedural complications. Two patients died during the first month (3.5%), with these two cases occurring 5 days following the procedure. In one of the cases, the death was due to cardiovascular reasons. Mortality in the second month was 5% and total mortality over 3-months was 16% (Table 2). There were no major bleeding events reported during hospitalization and follow-up. The mean age of patients who died was older than those who survived, but this difference was not significant (77.63 ± 11.83, and 70.77 ± 0.26, respectively). The rate of death was twofold higher in men (20%) than in women (11%). Mortality rate was higher in patients who suffered from stroke (44%) and in those with renal failure (44%, χ2 = 9.36, *p* = 0.002). We found no correlation between the mortality and the nutritional status before the insertion of PRG.

**Table 2:** Adverse events of the study patients during a 3-month follow-up period *N* = 58.

	**Follow up (n/% of patients)**		
	**1 month**	**2 months**	**3 months**
Death	2 (3%)	3 (5%)	4 (7%)
Peritonitis	2 (3%)	1 (2%)	
Aspiration	2 (3%)	1 (2%)	2 (3%)
Local infection	20 (35%)	11 (19%)	8 (14%)
Tube dislodgement	2 (3%)	7 (12%)	8 (14%)

A total of 64 post-procedure complications occurred in 39 patients (67% of all patients), or 1.64 per patient (Table 2). Of these, 88% were minor, with an overall rate of 1.51 complications per patient. In 17 (29%) patients more than 2 complications were reported, and in four (24%) of these patients four complications were reported. A serious complication occurred in 19 patients (33%). Peritonitis was the leading cause of death in 2 patients.

Tube dislodgement occurred in 17 subjects and was the main cause of all serious complications in about two-thirds of cases. Tube blockage occurred in 10 (17%) patients, but none of these required hospitalization or surgical intervention. Aspiration pneumonia was the second serious complication and was reported in 9% of all patients. No mortality was associated with aspiration pneumonia. The rate of local infection was relatively high. It was reported in 67% of all patients. Most of these were mild and responded to local antibiotic treatment. During the follow up period, 17 (29%) patients were re-admitted to the hospital. In half of all hospitalization cases, the cause of hospitalization was unrelated to the PRG procedure. The main cause of re-admission was dislodgement of the tube (22%), followed by peritonitis (17%) and aspiration pneumonia (11%). The changes in nutritional status on follow up are presented in Table 3.

**Table 3:** Changes in laboratory data over a 3-month period of follow-up.

	**Mean** ± **SD**
BMI* kg/m2	0.13 ± 4.07
Albumin g/dL	0.16 ± 0.47
Hemoglobin g/dL	0.34 ± 1.13
WBC^†^ cells/μL	0.43 ± 3.61

† *BMI, body mass index*,

† *WBC, white blood cells*.

## Discussion

We found that PRG is a reasonable option for enteral feeding in older patients. Guidelines suggest that enteral feeding should be considered if it is expected that a patient's nutritional intake is likely to be qualitatively or quantitatively inadequate for a period of more than 2 to 3 weeks. The maintenance of adequate nutrition remains an important goal in the management of many chronic conditions, including cerebrovascular and neurodegenerative diseases. Enteral feeding is the preferred route of nutrition in patients with a variety of conditions when gut function is maintained, as it provides greater immunological and nutritional benefits compared to parenteral feeding.

Gastrostomy is the preferred method for the administration of enteral nutrition in patients with impaired swallowing and dysphagia. Enteral access can also facilitate the delivery of medications in these patients who are unable to swallow (3, 6, 10, 11). Percutaneous gastrostomy techniques with either radiological or endoscopic guidance have generally replaced an open surgical approach, mainly because of the risk of general anesthesia and increased morbidity. While the endoscopic approach predominates, in this study we investigated PRG as an alternate approach.

While we found no immediate complications relating to the anesthesia or the procedure, subsequent complications were not uncommon. Although most adverse outcomes were minor, major complications did occur. Peritonitis led to one-fifth of the overall mortality. This complication is caused by either intraperitoneal leakage around the puncture site or from tube migration and erosion causing frank gastric perforation. While this complication is clearly tube-related, we speculate that overall mortality was probably multifactorial. The design of this observational prospective study limits our ability to determine whether mortality was in fact disease-related, which would concur with the literature (12). Older patients, such as those in our study, with severe neurological disorders and frailty, are more susceptible to complications related to the procedure and to adverse outcomes. Additionally, since age and male gender are the primary predictors of mortality in older patients, the observed mortality in our older predominantly male cohort was not unexpected.

Previous studies have reported that the incidence of bleeding complications after PRG or PEG were 0.7–3.0% (13). An important finding of our study is that no adverse events of bleeding were reported, despite not stopping anti-aggregate or anti-coagulant therapy. Aspiration pneumonia occurred in a small number of patients. According to a previous report (14), aspiration and aspiration pneumonia occurred less frequently with PRG than PEG, possibly because of the need for more sedation and the endoscopic technique used during PEG.

Tube dislodgement is a serious complication of the PRG procedure, especially if it occurs soon after the initial insertion, since it may lead to malposition and result in peritonitis. This is associated with higher costs and resource burden, due to the need for a repeat procedure and other interventions. The high rate of tube dislodgement in our study (29%) is consistent with other published studies which report dislodgement rates from 21 to 30% (15–18). The higher rate of dislodgement with PRG was independent of the patient's age (17). In our study, in all causes of dislodgement, tubes were repositioned or replaced with a wider diameter catheter.

Blockage of the tube is a frequent problem observed in patients with long-term enteral feeding. Blacka et al. (19) reported that with regard to patients with PEG, from 16% to 31% of tubes had at least 1 episode of significant blockage during 18 months of follow-up, and 7% required removal. This rate is generally higher in PRG because of the smaller diameter of the tube (20). In our study, tube blockage occurred in 17% of the patients, and none of them was substantial. They were all treated in the geriatric department and none of them needed surgical intervention. In addition, where necessary we subsequently replaced the tube with a Foley-catheter with a wider diameter to prevent blockage of the tube. In one case of a partially blocked tube tract, the blockage was treated by washing with a saline 0.9% solution.

Deep infection of the stoma is more frequent in PEG than in PRG (18, 20), because the PEG tube is inserted through the mouth and oropharynx, where the risk of contamination by the oral flora is higher, leading to wound infections. Prophylactic antibiotics is required in PEG insertion. However, prophylactic antibiotics is not routine practice in PRG insertion. In our study this complication was not found.

When comparing the overall numbers of episodes of minor complications with the results documented in the literature, the rates are similar (3, 14, 21–23). Similarly, results regarding the number of people who had minor complications are comparable. In our study, all causes of local infections were superficial.

## Conclusion

Our study suggests that PRG is relatively safe and effective for gastrostomy placement in older patients requiring long-term enteral nutrition, with adverse outcomes being similar to those of PEG. The absence of bleeding complications secondary to PRG insertion despite ongoing anti-aggregate and anti-coagulant treatment suggests a possible advantage to this technique. Furthermore, since PRG uses a sterile technique and does not involve passing a scope via the oropharynx, the fact that none of our patients developed deep wound infection at the site of the stoma should be noted. Based on the findings of this relatively small observational study, further research is required to compare this technique to the endoscopic approach.

## Author contributions

All authors listed have made a substantial, direct and intellectual contribution to the work, and approved it for publication.

### Conflict of interest statement

The authors declare that the research was conducted in the absence of any commercial or financial relationships that could be construed as a potential conflict of interest. The handling Editor declared a shared affiliation, though no other collaboration, with several of the authors VH, YK (RD), YK (MD), IK, DMN, and GM.
